# Focused Ultrasound-Induced Cavitation Sensitizes Cancer Cells to Radiation Therapy and Hyperthermia

**DOI:** 10.3390/cells9122595

**Published:** 2020-12-03

**Authors:** Shaonan Hu, Xinrui Zhang, Michael Unger, Ina Patties, Andreas Melzer, Lisa Landgraf

**Affiliations:** 1 Innovation Center Computer Assisted Surgery (ICCAS), University of Leipzig, 04103 Leipzig, Germany; shaonan.hu@medizin.uni-leipzig.de (S.H.); michael.unger@medizin.uni-leipzig.de (M.U.); ina.patties@medizin.uni-leipzig.de (I.P.); lisa.landgraf87@gmail.com (L.L.); 2 Department of Radiation Oncology, University of Leipzig, 04103 Leipzig, Germany; 3 Institute for Medical Science and Technology (IMSaT), University of Dundee, Dundee DD2 1FD, UK

**Keywords:** focused ultrasound, cavitation, radiation therapy, hyperthermia, sensitization

## Abstract

Focused ultrasound (FUS) has become an important non-invasive therapy for solid tumor ablation via thermal effects. The cavitation effect induced by FUS is thereby avoided but applied for lithotripsy, support drug delivery and the induction of blood vessel destruction for cancer therapy. In this study, head and neck cancer (FaDu), glioblastoma (T98G), and prostate cancer (PC-3) cells were exposed to FUS by using an in vitro FUS system followed by single-dose X-ray radiation therapy (RT) or water bath hyperthermia (HT). Sensitization effects of short FUS shots with cavitation (FUS-Cav) or without cavitation (FUS) to RT or HT (45 °C, 30 min) were evaluated. FUS-Cav significantly increases the sensitivity of cancer cells to RT and HT by reducing long-term clonogenic survival, short-term cell metabolic activity, cell invasion, and induction of sonoporation. Our results demonstrated that short FUS treatment with cavitation has good potential to sensitize cancer cells to RT and HT non-invasively.

## 1. Introduction

Decades of intensive research and technology development on the therapeutic applications of focused ultrasound (FUS), also described as high-intensity focused ultrasound (HIFU) usually used for thermal tissue ablation, have led to the clinical approval for the treatment of prostate cancer, uterine fibroids, essential tremor, and pain release of bone metastases [1]. Compared to conventional hyperthermia methods, FUS-induced heating can be focused within pathological tissue in a region of a diameter of only 2 mm [2]. Moreover, due to magnetic resonance (MR) imaging guidance, precise treatment planning and non-invasive real-time temperature monitoring via MR thermometry are feasible.

Conventional hyperthermia (HT) has been reported in many preclinical and clinical studies to sensitize especially hypoxic tumors to radiation therapy (RT) via tumor oxygenation and DNA repair inhibition [3,4]. HT techniques can be categorized into whole body HT, local HT, and regional HT, which are all typically applied to treat metastasis, localized solid tumors, and cancer diseases in deeper tissue, respectively [5]. In clinical use, the heating is mainly delivered by electromagnetic waves, e.g., micro- and radiofrequency waves. Other HT methods, such as magnetic nanoparticle heating, are still in the developmental stage [6]. The precise delivery of heat to deep local tumors without damaging the surrounding tissue is still the main challenge.

The modes of action of FUS to induce cellular destruction are classified into thermal and mechanical effects [7]. One of the most essential mechanical effects in the therapeutic ultrasound field is cavitation, which can be described as oscillation and collapse of the gas-filled cavities in the acoustic field. These processes are known as stable (non-inertial) and inertial cavitation [8]. Stable cavitation close to the cell membrane leads to an increase in membrane permeability [9]. This process is described as sonoporation and has been reported to support the delivery of therapeutic agents from drug carriers through the cell membrane by the effect of stable cavitation [10,11,12]. In contrast, the blood–brain barrier (BBB) can also be opened via FUS by temporary loosening of the tight junctions between endothelial cells allowing the delivery of therapeutic drugs to the central nervous system [13]. Contrastingly, inertial cavitation can induce direct mechanical tissue damage applied as lithotripsy in the destruction of, e.g., gallstones [14]. Furthermore, cavitation was also reported to enhance treatment effects during ablation and mediates in situ drug or gene delivery by increasing temperatures and the induction of mechanical damage [15,16].

Many groups have investigated the quantification for the monitoring and controlling of cavitation, for instance, laser-induced cavitation has been developed for better understanding the process [17]. A commonly applied method is the integration of a passive cavitation detector (PCD) using a single-element focused transducer or a hydrophone as a receiver which listens to acoustic emissions from cavitation bubbles [18,19,20]. Hereby, emissions at sub- and ultra-harmonic of the drive frequency and broadband noise are widely accepted as indicators of stable and inertial cavitation in the literature, respectively [8]. Additionally, inertial cavitation can be evaluated chemically with terephthalate acid (TA) by assessing the generation of free radicals. FUS-induced inertial cavitation leads to water sonolysis and the formation of hydroxyl radicals (·OH) and hydrogen radicals (·H). The TA is used as a dosimetric solution because it is oxidized by hydroxyl radicals during sonication leading to the production of the fluorescent 2-hydroxylterephthalic acid, which can be quantified by using fluorescence spectroscopy indirectly representing the inertial cavitation dose under laboratory conditions in vitro [21].

As mentioned above, the cavitation effect is typically applied for the delivery of therapeutic agents so far, cavitation-based antivascular effects were also reported in vivo [22]. However, the effect of cavitation in combination with other therapies has not been sufficiently investigated due to poor controllability during treatment with clinical approved MR-guided FUS. Moreover, a safe, efficient, and non-toxic adjuvant therapy to conventional therapies, especially RT and HT, is necessary to reduce adverse effects while increasing the treatment outcome in clinical practice. Therefore, we investigated in this study the potential of short FUS shots with (FUS-Cav), or without cavitation (FUS) as a sensitizer to RT and HT with an original in vitro FUS system in three different tumor cell culture model. A fiber-optic hydrophone (FOH) was used to quantify the cavitation dose inside the 96-well plate. The biological effects of FUS in combination with RT or HT were evaluated by the long-term reproductive cell survival, cellular metabolic activity, and cell invasion in vitro.

## 2. Materials and Methods

### 2.1. Tumor Cell Lines and Cell Culturing

The human head and neck cancer cell line FaDu (OncoRay, National Center for Radiation Research in Oncology, Dresden, Germany) was cultured in Dulbecco’s Modified Eagle’s Medium (DMEM, Gibco, Thermo Fisher Scientific, Dreieich, Germany) supplemented with 2% HEPES (1 M, PAA Laboratories), 1% sodium pyruvate (100 mM, Sigma-Aldrich GmbH, Munich, Germany), and 1% MEM non-essential amino acids (100×, Sigma-Aldrich GmbH, Munich, Germany). The human glioblastoma cell line T98G, obtained from Prof. Gaunitz (Department of Neurosurgery, University of Leipzig, Leipzig, Germany), was cultured in DMEM. The human prostate cancer cell line PC-3, purchased from the European Collection of Authenticated Cell Cultures (ECACC, Salisbury, UK), was cultured in Ham’s F-12K (Kaighn’s) medium (Gibco, Thermo Fisher Scientific, Dreieich, Germany). All cell culture mediums were supplemented with 10% (*v/v*) fetal bovine serum (FBS, Gibco, Thermo Fisher Scientific, Dreieich, Germany), 100 U/mL penicillin, and 100 mg/mL streptomycin (Biochrom GmbH, Berlin, Germany) and cells were cultivated at 37 °C with 5% (*v*/*v*) CO_2_ in humidified air. For experiments, cells were routinely washed with phosphate-buffered saline (PBS) without Ca^+^, Mg^+^, and phenol red (Biozym Scientific GmbH, Hessisch Oldendorf, Germany) and detached using trypsin/EDTA (Biozym Scientific GmbH, Hessisch Oldendorf, Germany). Cells were routinely tested for mycoplasma.

### 2.2. FUS In Vitro System

An experimental in vitro FUS device composed of a water tank, a 3D-printed cell culture well plate holder, a stepper motor (all VELMEX Inc., Bloomfield, NY, USA) to move the plate across the transducer, and a self-priming pump (Lei Te Co., Ltd., Guangdong, China) circulating the water to avoid bubble formation and overheating, was used. As an FUS source, interchangeable customized single-element transducers with a frequency of 1.467 MHz were used. The system was applied for the treatment of multi-wells in 96-well plate as described in detail previously [23]. Briefly, the transducer emits a continuous wave with a signal generator (33120A, Agilent Technologies, Edinburgh, UK) and a radiofrequency power amplifier (A075, Electronics and Innovation, Rochester, NY, USA) to create a wide range of highly reproducible FUS conditions. Intensities ranging from 129 to 1704 W/cm^2^ were applied to induce mechanical effect and cavitation. The transducers were calibrated with a fiber-optic hydrophone (FOH, Precision Acoustics, Dorchester, UK). A thermal camera (Optris PI450, Optris GmbH, Berlin, Germany) was mounted to detect the real-time temperature in the wells. Ultrasound-penetrable 96-well µclear^®^ cell culture plates (Greiner Bio-One, Frickenhausen, Germany) were used for the treatment of cancer cells in vitro.

### 2.3. Cavitation Dose Measurement with a Fiber-Optic Hydrophone (FOH)

**Figure 1:**
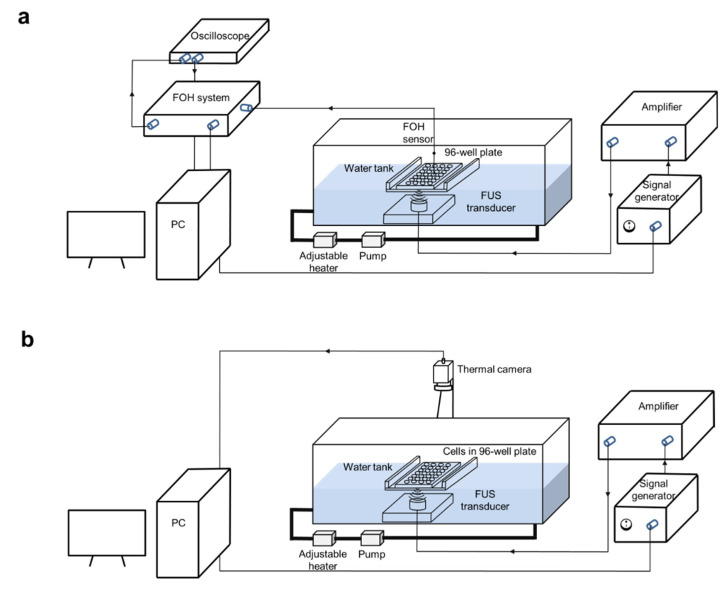
Experimental setup for cavitation detection and focused ultrasound (FUS) treatment. The in vitro FUS system includes a signal generator (Agilent 33120A), a radio frequency (RF) power amplifier (Electronics and Innovation A075), a customized FUS transducer and a water tank with adjustable heater and pump. (**a**) For the cavitation measurement, a 96-well plate filled with water was immersed in the water tank, the fiber-optic hydrophone (FOH) sensor was placed close to the bottom of the plate at the focal point acquiring the cavitation signal with oscilloscope. (**b**) FUS treatment was performed with monolayer cancer cells in a 96-well plate, the plate was sealed with water-proofed film to keep cells in a sterilized environment, a thermal camera (Optris PI450) was utilized for real-time temperature monitoring. The FUS treatment was controlled with a feedback loop to the thermal camera using LabView program.

In the first step, the cavitation signal was acquired via the FOH system. It consists of an optical fiber sensor with an outer diameter of 125 µm and a sensitivity of 138 mV/MPa at 1.467 MHz. The FOH sensor was positioned inside the wells filled with 420 µL deionized water and the focal spot at the bottom of the well (Figure 1a). The detected signals were measured by an oscilloscope (PicoScope 5243B, Pico Technology, St Neots Cambridge Shire, UK), which was controlled by a LabView program (National Instruments, UK) for transferring and storing the data. The signal was analyzed in Matlab (MathWorks, Natick, MA, USA). Therefore, a set of 67,000,000 samples at 20 MS/s (mega sample per second) was acquired synchronously to the sonication. The data were processed by (1) splitting the binary data of each signal into 625 segments per second, (2) fast Fourier transformation (FFT) of each segment, and (3) calculating the total root-mean-square (RMS) voltage of the signal over a chosen range of frequencies at a single time point (RMS voltage***_t_***). Stable cavitation was defined based on the sub- and ultra-harmonic signals (m × f_0_ /2, f_0_: fundamental frequency, m = 1, 3, 5, 7 …) in the frequency spectrum. A bandwidth of ± 20 kHz of sub- and ultra-harmonics was chosen as the frequency range for the calculation of the RMS voltage***_t_***. The inertial cavitation was defined based on the frequency spectrum after excluding the fundamental harmonics, sub- and ultra-harmonic signals. The RMS voltage***_t_*** was plotted as a function of sonication time. Due to the limited storage of the oscilloscope, the total cavitation dose for 40 s was measured as a sum up of 14 consecutive FUS periods of 2.9 s. The cavitation dose for each single period of 2.9 s was defined as the integral of RMS voltage***_t_*** over time with baseline noise removed: where t is the time for each sonication segment (1.6 ms); T is the sonication period (2.9 s); RMS voltage_t_ is the cavitation level of one sonication segment for 1.6 ms.

(1) $$\begin{array}{c} \mathrm{Cavitation}\;\mathrm{dose}\;\left(2.9\;s\right)\;=\;\sum_{t\;=\;0\;-\;T}\mathrm{dRMS}\;{\mathrm{voltage}}_{t}(\mathrm{FUS})\\ -\;\sum_{t\;=\;0\;-\;T}\mathrm{dRMS}\;{\mathrm{voltage}}_{t}(\mathrm{Background}\;\mathrm{noise}) \end{array}$$

### 2.4. Cavitation Dose Measurement with Terephthalic Acid (TA)

To validate the measurement of the hydrophone, inertial cavitation dose was measured in the 96-well plate format via a chemical method using TA (Sigma-Aldrich GmbH, Munich, Germany) according to Barati et al. [24]. The 2 mM TA solution was prepared by dissolving 0.0831 g TA powder and 0.05 g NaOH (Sigma-Aldrich GmbH, Munich, Germany) with 250 mL pre-warmed PBS. Up to 420 μL/well of TA solution was added in the plate and sealed with Titer-tops (Sigma-Aldrich GmbH, Munich, Germany) plate-sized US transparent films, avoiding air bubble formation. Acoustic intensities at 129, 344, 539, 1136, and 1704 W/cm^2^ were applied with a sonication duration of 40 s. Afterward, the plate was incubated in darkness at room temperature for 3 h. A total of 200 μL TA sample solution was transferred from the treated plate to a new black 96-well plate (Greiner Bio-One, Frickenhausen, Germany) and fluorescence intensity was measured by a plate reader (Synergy H1, BioTek, Bad Friedrichshall, Germany) with excitation/emission wavelengths at 310/425 nm.

### 2.5. FUS Treatment of Cancer Cells

Cells were seeded at a density of 2500–10,000 cells/well in corresponding cell culture medium to reach 80–100% confluency at the desired time point after treatment. The seeding was performed 24 h before the treatment. Up to 420 μL/well of cell culture medium was added in each well to the attached cells and the plate was sealed with Titer-tops film before sonication. To separate the thermal and mechanical effect of FUS, an infrared thermal camera was used to monitor the temperature of the wells (Figure 1b). Once the temperature in the well reached 39 °C, the sonication was automatically stopped until the temperature decreased to the baseline of 34 °C. Two different treatment protocols were conducted using continuous wave ultrasound with monolayer cells in a 96-well µclear^®^ plate as follows: (1) short FUS treatment (FUS) with an acoustic intensity of 129 W/cm^2^ and an active sonication duration of 40 s. (2) FUS-induced cavitation (FUS-Cav) with acoustic intensity at 1136 W/cm^2^ and an active sonication duration of 40 s.

### 2.6. HT Treatment with Water Bath

Cells were seeded in a 96-well plate at the density of 2500–10,000 cells/well in the corresponding cell culture medium 24 h before the HT treatment. The plate was sealed with Titer-tops film before HT treatment and placed in a pre-warmed water bath (GFL Gesellschaft für Labortechnik mbH, Burgwedel, Germany). The thermocouples (OMEGA, Manchester, UK) were inserted into two reference wells and Pico Log (Pico Technology, St Neots Cambridge Shire, UK) was used to measure real-time temperatures and collect data. HT treatment of cells was performed at 45 °C for 15, 30 and 60 min with a water bath, and, based on a preliminary experiment, HT at 45 °C for 30 min was chosen for further experiments (Figure S1).

### 2.7. RT with X-ray In Vitro

Cells cultured in the 96-well plates were irradiated with a single dose using an X-ray machine (XStrahl 200, XStrahl, Camberley, UK) at a dose rate of 1.07 Gy/min. For determination of the non-lethal radiation dose, radiation dose curves with 0 to 20 Gy were obtained. Based on previous data, clinical-relevant single irradiation doses of 5 and 10 Gy [25,26] were chosen for the combination experiments.

### 2.8. Combination Treatment Protocol of Cancer Cells

**Figure 2:**
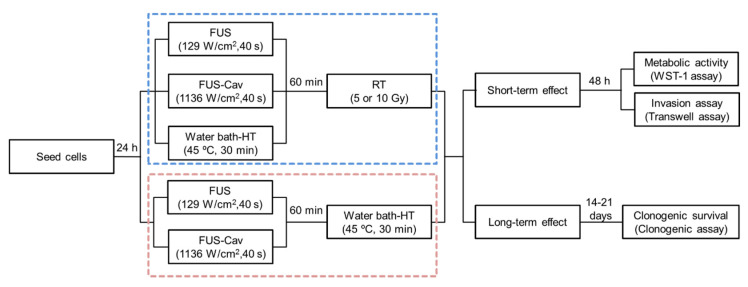
Experimental timeline describing the procedure of the combination treatments and biological assays. The combination treatment of FUS/FUS-Cav/ hyperthermia (HT) and radiation therapy (RT) is shown in the blue square, and the treatment process of the combination of FUS/FUS-Cav and HT is shown in the red square.

The cells were seeded at densities of 2500–10,000 cells/well in corresponding culture medium 24 h before treatment allowing formation of monolayer. To investigate the additive effects of FUS/FUS-Cav to RT, the cancer cells were first exposed to FUS/FUS-Cav according to the single treatment protocol, and water bath HT was conducted as a reference. RT treatment was conducted with an interval time of 60 min after single FUS/FUS-Cav or HT (Figure 2 blue square). The combination treatment of FUS/FUS-Cav and HT was performed as shown in Figure 2 (red square). The short-term effect was evaluated after 48 h by measurement of metabolic activity and cell invasion, the long-term effect was assessed by clonogenic assay 14–21 days post-treatment.

### 2.9. Clonogenic Assay

To examine the long-term survival of clonogenic cells after different treatment regimes, clonogenic assay was performed as described previously [27]. Briefly, cells were treated in 96-well plates as described above, and the untreated group and single RT at 5 or 10 Gy were used as a control. Cells were harvested by trypsin/EDTA after treatment and seeded in triplicates at a density of 200–10,000 cells/well into 6-well plates (Greiner Bio-One GmbH, Frickenhausen, Germany). Plates were incubated for 14–21 days (depending on cell line) to allow colony formation, the culture medium was changed twice per week. Colonies were gently rinsed with PBS twice before being fixed with ice-cold ethanol/acetone (1:1, *v*/*v*) for 5 min, stained with 0.5% crystal violet (Sigma-Aldrich GmbH, Munich, Germany) solution in water for 30 min and washed with distilled water to remove unbound stain. Colonies in dried plates were scored if they exceeded a threshold number of 50 cells. The plating efficiency is defined as the number of surviving colonies divided by the number of cells seeded. To obtain the cell clonogenic survival fraction (SF), the ratio between plating efficiency of treated cells and plating efficiency of untreated cells was calculated [28].

### 2.10. WST-1 Assay

To determine the impact of the different treatments on the cellular metabolic activity of the human cancer cell lines, the tetrazolium salt-based metabolic activity assay WST-1 (Roche, Basel, Switzerland) was performed 48 h after therapy, and untreated cells were used as a control. According to the manufacturer’s instructions, the culture medium was discarded and cells were incubated with fresh cell culture medium including WST-1 reagent (final concentration of 10%) in the 96-well cell culture plates. The absorbance at 435 nm was measured using a plate reader (Synergy H1, BioTek, Bad Friedrichshall, Germany).

### 2.11. Invasion Assay

To evaluate the potential of tumor cells to invade, the in vitro Transwell^®^ invasion assay was performed [29] with untreated cells as a control. The Transwell^®^ chambers (Corning Inc., New York, NY, USA) with the polycarbonate membrane were coated with 100 µg/cm^2^ Matrigel (Corning Inc., New York, NY, USA) at 37 °C for 4 h, and were mounted on a 24-well plate (Sigma-Aldrich GmbH, Munich, Germany) as the upper chamber. The prostate cancer PC-3 cells were harvested from 96-well plates after treatment, and 1 × 10^5^ cells were suspended with 100 μL serum-free medium and seeded in the upper Transwell^®^ chamber. Complete medium supplemented with 10% FBS was added into the lower chamber as a source of chemotactic factor. The 24-well plate with Transwell^®^ chambers was incubated at 37 °C for 48 h. The non-invaded cells at the upper surface of the membrane were scraped off with cotton swab, and the remaining cells at the lower surface of Transwell^®^ chamber were fixed with methanol (Carl-Roth, Karlsruhe, Germany) and stained with 0.1% crystal violet. Invaded cells were observed under a microscope (ZEISS Axio Observer, Carl Zeiss Microscopy GmbH, Jena, Germany) and 5 random fields at 200-fold magnification in each chamber were selected for cell counting using ImageJ software.4.12.

### 2.12. Detection of Sonoporation by Cell Staining with Propidium Iodide (PI)

Sonoporation is defined as the transfer of extracellular molecules into a cell generally induced by stable cavitation [30]. To investigate this phenomenon on cells, PI was employed as a cell membrane integrity probe that cannot penetrate through intact cell membranes of living cells and CellMask^TM^ Green Plasma Membrane Stain (Thermo Fisher Scientific, Dreieich, Germany) was used for visualization of the cell membrane. Cells were seeded in a 96-well plate at a density of 5000 cells/well 24 h before treatment. FUS-Cav treatment was conducted as described above and cells were washed gently with PBS afterward. PI at a final concentration of 1 µg/mL and CellMask^TM^ Green Plasma Membrane Stain at a final concentration of 5 µg/mL were added in cell culture medium before or 30 min after sonication of the cells. Immediately, PI was visualized at excitation/emission at 535/617 nm and cell plasma membrane stained with CellMask^TM^ at ex 522/ em 535 nm at 200-fold magnification using a fluorescence microscope and ZEN 2.3 software (ZEISS Axio Observer, Carl Zeiss microscopy GmbH, Jena, Germany). Five random fields were chosen for observation, and the cells stained with PI and CellMask^TM^ (approximately 150 cells in each field) were counted to quantify the percentage of PI-positive cells.

### 2.13. Statistical Analysis

The cavitation dose was measured at each intensity of nine independent exposures. All data of metabolic activity (WST-1 assay), survival fractions (clonogenic assay), cell invasion (transwell assay), and sonoporation efficiency (PI uptake assay) were expressed as means ± SEM (Standard error of the mean) of three independent experiments in two replicates, respectively. The significance of the difference between the two mean values was assessed by a one-way ANOVA test and Tukey test for post-hoc analysis. Statistical analysis of the clonogenic survival data was performed using non-parametric Mann–Whitney test in SPSS statistic software version 24. A *p*-value ≤  0.05 was considered to be statistically significant.

## 3. Results

### 3.1. Cavitation Occurs at a Certain Level of Intensity

**Figure 3:**
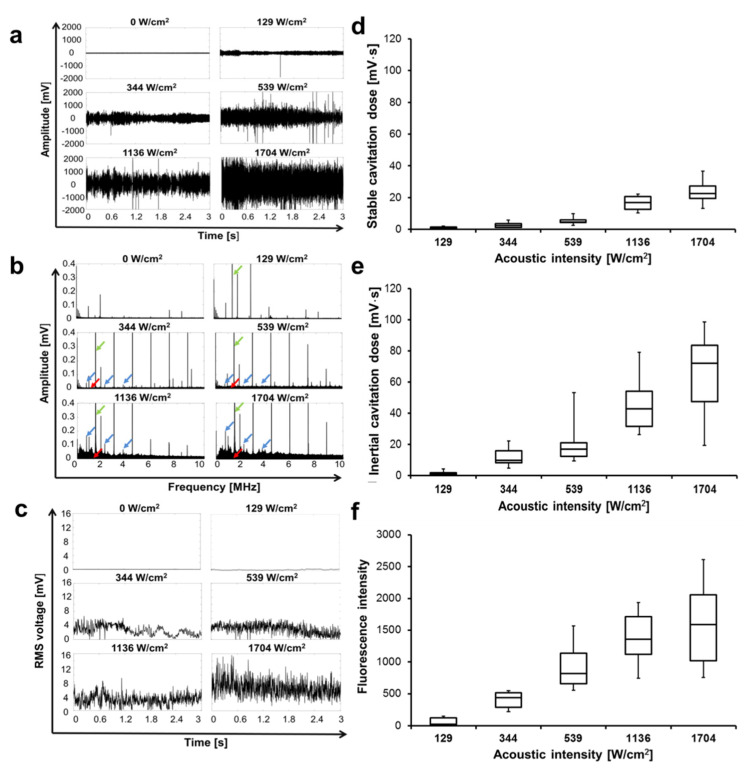
Representative acoustic emission signals at 1.467 MHz and influence of acoustic intensity on the type and dose of cavitation. (**a**) The time-domain plots of the acoustic signals for one recording period of 2.9 s. (**b**) Corresponding frequency-domain plots; green arrow: fundamental frequency (f_0_) 1.467 MHz; blue arrow: sub- and ultra-harmonics (f = m × f_0_ /2, m = 1, 3, 5…) represent stable cavitation; red arrow: broadband noise represent inertial cavitation. (**c**) Root-mean-square (RMS) voltage of the broadband noise as a function of exposure time. (**d**) Stable and (**e**) inertial cavitation dose with a sonication duration of 40 s measured with a fiber-optic hydrophone, n = 9. (**f**) Fluorescence intensity measured by the terephthalic acid (TA) method indicates an inertial cavitation dose with a sonication duration of 40 s, n = 9.

The spectrograms with various acoustic intensities are shown in Figure 3. Time domains represent the acoustic amplitude changes in time series (Figure 3a), the amplitude increased with increasing acoustic intensity. Stable and inertial cavitation is represented by sub- and ultra-harmonics signals and broadband noise, respectively. Both cavitation signals are visualized in Figure 3b with an acoustic intensity (temporal peak intensity) above 129 W/cm^2^. The RMS voltage of each segment is shown in Figure 3c over a sonication period of 2.9 s, and the integral of the area under the RMS voltage–time curve was defined as the cavitation dose. Both of the inertial cavitation dose and stable cavitation dose (Figure 3d–f) directly correlate with acoustic intensity. The sub- and ultra-harmonic signals (m × f_0_ /2, f_0_: fundamental frequency, m = 1, 3, 5, 7…) represent stable cavitation, the stable cavitation dose was 0.90 ± 0.65 mV⋅s at an intensity of 129 W/cm^2^ with a total sonication duration of 40 s and was significantly enhanced to 16.33 ± 4.29 mV⋅s when the intensity was increased to 1136 W/cm^2^. A 1.5-times higher stable and inertial cavitation dose could be obtained at 1704 W/cm^2^ in comparison to 1136 W/cm^2^.

The inertial cavitation threshold was defined as the minimum negative pressure of broadband noise at which any signal greater than three times of standard deviation (SD) of background noise occurred. In our study, the SD of the background noise of the unsonicated samples was 2.07 mV⋅s (data not shown). Hydrophone measured the inertial cavitation dose of 0.82 ± 1.25 mV⋅s after exposure with FUS at the intensity of 129 W/cm^2^ and inertial cavitation dose of 11.82 ± 5.48 mV⋅s at the intensity of 344 W/cm^2^. The results of inertial cavitation were confirmed with the TA method by measuring fluorescence intensity (Figure 3f). The trend of fluorescence intensity was comparable to the inertial cavitation dose results measured by the hydrophone. In this context, the inertial cavitation occurred at acoustic intensities of 344 W/cm^2^ and above. Based on the cavitation measurement and threshold definition, the FUS treatment was defined as short FUS shots without inertial cavitation at an intensity of 129 W/cm^2^ and FUS-Cav treatment with cavitation at an intensity of 1136 W/cm^2^.

### 3.2. Short High-Intensity Cavitation-Inducing FUS Shots (FUS-Cav) Are Effective to Radiosensitize Tumor Cells

**Figure 4:**
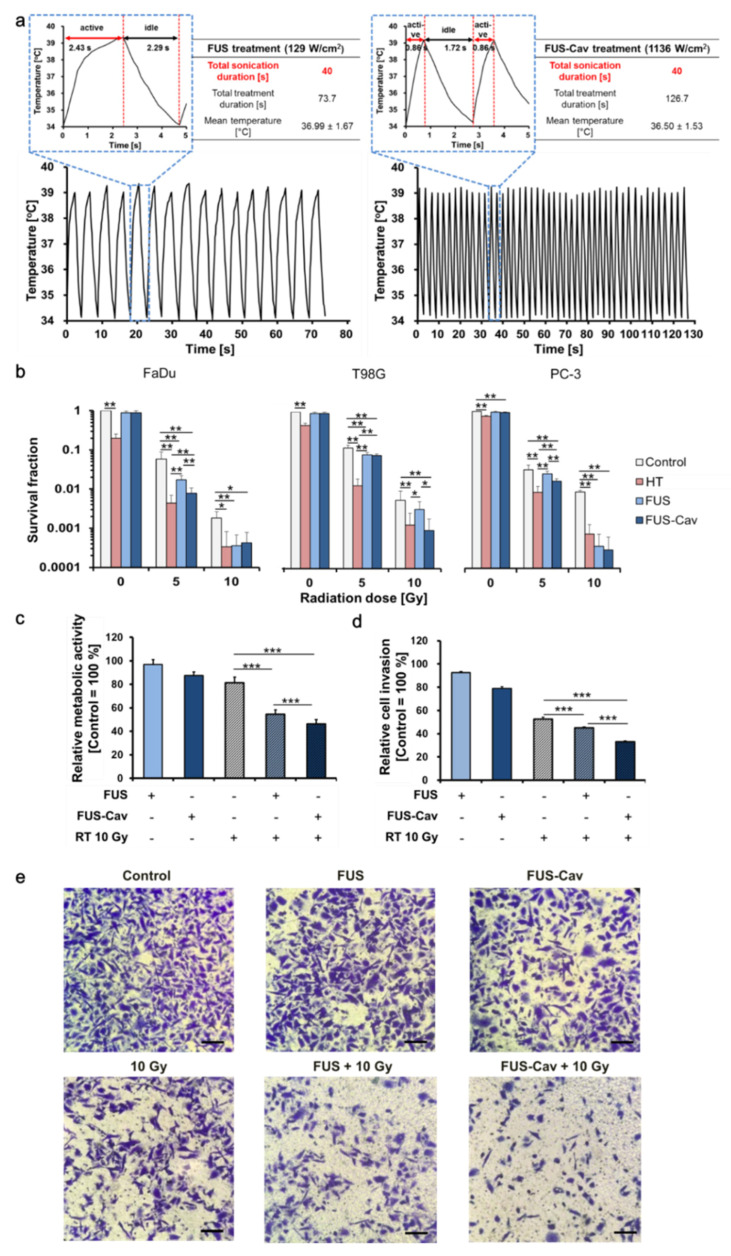
FUS (129 W/cm^2^, 40 s) and FUS-Cav (1136 W/cm^2^, 40 s) revealed a radioadditive effect on reproductive long-term survival, metabolic activity and cell invasion. (**a**) Temperature curves of FUS and FUS-Cav treatment with a mean temperature of 36.99 ± 1.67 °C and 36.50 ± 1.53 °C. The diagram illustrated the sonication active duration (red arrow) and idle (black arrow) duration in each cycle, and the total treatment duration of FUS and FUS-Cav is 73.7 s and 126.7 s, respectively. (**b**) The clonogenic survival of FaDu, T98G and PC-3 cells was evaluated after FUS and FUS-Cav treatment in combination with radiation at a single dose of 5 or 10 Gy. Water bath HT (45 °C, 30 min) was performed as a reference. (**c**) Relative cell metabolic activity of PC-3 cells measured with WST-1 assay and (**d**) semi-quantitative result of the Transwell^®^ assay indicating cell invasive potential of PC-3 cells 48 h post-treatment. Data were normalized to untreated control, which were set as 100% and relative values presented as mean ± standard error of the mean (SEM), n = 6, * *p* ≤ 0.05; ** *p* ≤ 0.01; *** *p* ≤ 0.001. (**e**) Representative microscopy images of Transwell^®^ assay in PC-3 cells 48 h post-treatment. Scale bar = 100 µm.

To control the temperature in a specific range and make the results comparable, the cells were exposed to ultrasound at different intensities in on/off mode with the same sonication duration of 40 s in FUS and FUS-Cav treatment. The temperature curves (Figure 4a) illustrated the different heating profile at two intensities, the temperature fluctuation was observed in both FUS and FUS-Cav treatment. The temperature increased to 39 °C when sonication was activated, then sonication was deactivated and resulted in a temperature decrease to 34 °C. For FUS treatment at acoustic intensity of 129 W/cm^2^, the sonication active duration of each cycle is 2.43 s and the whole treatment duration is 73.7 s with the mean temperature of 36.99 ± 1.67 °C. While in FUS-Cav treatment at an intensity of 1136 W/cm^2^, sonication active duration of each cycle is 0.86 s and the treatment duration is 126.7 s with the temperature of 36.50 ± 1.53 °C.

Short FUS treatment alone had no impact on the reproductive survival of all cell lines (Figure 4b); in contrast, single HT treatment at 45 °C for 30 min resulted in a reduction in clonogenic survival compared to the untreated control. Interestingly, combined with RT, short FUS-Cav displayed comparable radioadditive effects such as HT for 30 min, especially in head and neck cancer FaDu cells and prostate cancer PC-3 cells. The clonogenic survival of FaDu cells was significantly decreased after the combination treatment of FUS/ FUS-Cav and RT (5 Gy) compared to RT alone. Combined treatments showed a 5.9-fold (HT + 10 Gy), 5.6-fold (FUS + 10 Gy), 4.7-fold (FUS-Cav + 10 Gy) survival fraction (SF) reduction compared to RT (10 Gy) in FaDu cells. In contrast, T98G cells revealed a different reaction where only FUS-Cav showed a significant radioadditive effect, and the water bath HT showed better radiosensitization effects compared to FUS and FUS-Cav at an RT dose of 5 Gy. Surprisingly, combination of FUS + 10 Gy (26-fold) and FUS-Cav + 10 Gy (32-fold) displayed a dramatically higher decrease in SF compared to HT + 10 Gy (9-fold) in PC-3 cells.

WST-1 assay and Transwell^®^ invasion assay were performed to evaluate the short-term radiosensitization effect of FUS or FUS-Cav treatments on PC-3 cell metabolic activity and cell invasion. The results show that cell metabolic activity and cell invasion of PC-3 cells were slightly reduced 48 h after short FUS and FUS-Cav alone (Figure 4c–e). Interestingly, the impact of RT was significantly enhanced by adding short FUS or FUS-Cav treatment. The combination of short FUS or FUS-Cav and RT (10 Gy) led to a significant loss of metabolic activity to 54.70 ± 3.58% (FUS + 10 Gy) and 46.51 ± 3.61% (FUS-Cav + 10 Gy) in comparison to single RT (10 Gy) (81.53 ± 4.62%). The cell invasion of PC-3 was reduced to 45.18 ± 0.74% (FUS + 10 Gy) and 33.35 ± 0.60% (FUS-Cav + 10 Gy) compared to single treatments (FUS: 92.69 ± 0.98%; FUS-Cav: 78.80 ± 1.62%; RT at 10 Gy: 52.82 ± 1.31%) 48 h after treatment.

### 3.3. Short High-Intensity FUS-Induced Cavitation Shots (FUS-Cav) Increase the Effect of HT

**Figure 5:**
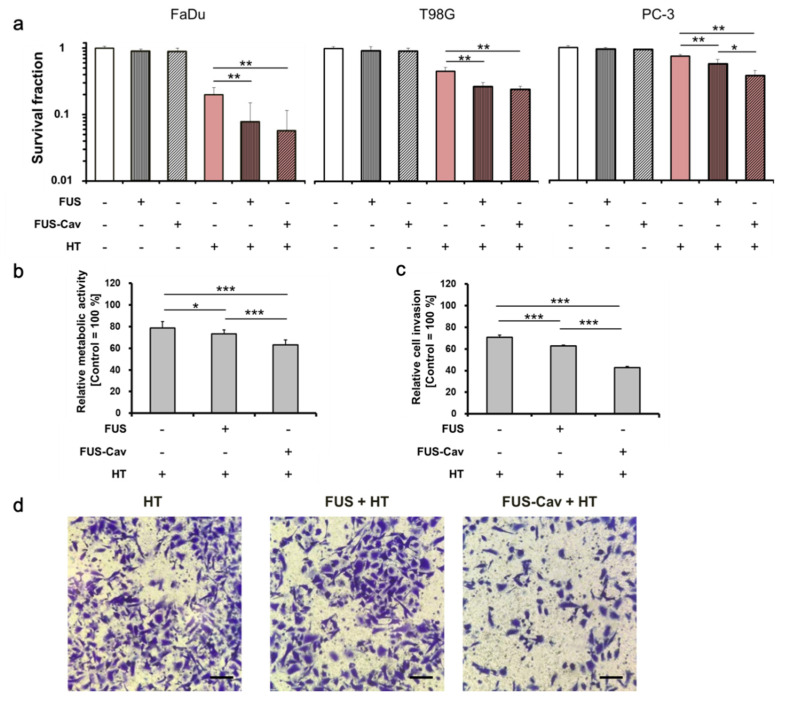
FUS (129 W/cm^2^, 40 s) or FUS-Cav (1136 W/cm^2^, 40 s) demonstrated the additive effect to HT (45 °C, 30 min). (**a**) Clonogenic survival of FaDu, T98G, and PC-3 cells was evaluated after HT, FUS, and FUS-Cav treatment. (**b**) Relative cell metabolic activity of PC-3 cells measured with WST-1 assay 48 h post-treatment. (**c**) Semi-quantitative result of the Transwell^®^ assay indicating cell invasive potential of PC-3 cells. (**d**) Representative microscopy images of Transwell^®^ assay 48 h post-treatment. Scale bar = 100 µm. Data were normalized to the untreated control, which were set as 100% and relative values presented as mean ± SEM, n = 6, * *p* ≤ 0.05; ** *p* ≤ 0.01; *** *p* ≤ 0.001.

To investigate whether short FUS sonication has additional benefits to HT, clonogenic survival of cells was evaluated after treatment. A reduction in SF was observed in all cell lines after the combination of short FUS or FUS-Cav and HT compared to single treatment groups. The effect on clonogenic survival after HT (SF: 0.20 ± 0.054 in FaDu, 0.45 ± 0.071 in T98G, 0.74 ± 0.042 in PC-3) was increased by adding short FUS (FUS + HT) showing a reduction of SF to 0.078 ± 0.073 in FaDu, 0.27 ± 0.043 in T98G, 0.57 ± 0.093 in PC-3 (Figure 5a). Moreover, an increased effect was seen by adding FUS-Cav to HT resulting in a 3.3-fold (SF: 0.06 ± 0.059 in FaDu), 1.9-fold (SF: 0.24 ± 0.030 in T98G), and 2-fold (SF: 0.37 ± 0.080 in PC-3) reduction in clonogenic survival compared to single HT treatment.

The effect on metabolic activity and invasion after combined treatment with short FUS or FUS-Cav and HT was evaluated in PC-3 cells only 48 h after treatment. Similar to the clonogenic assay experiments, short FUS and FUS-Cav showed significant additional benefits on the reduction of cell metabolic activity and invasion (Figure 5b–d). Thereby, cavitation effect enlarged the impacts of combination treatment of FUS-Cav + HT reducing metabolic activity from 87.46 ± 3.18% (FUS-Cav) and 78.79 ± 5.89% (HT) to 62.98 ± 4.74% (FUS-Cav + HT). The potential of cells to invade decreased from 70.73 ± 2.14% (HT) to 62.95 ± 0.66% (FUS + HT) and 42.67 ± 1.17% (FUS-Cav + HT).

### 3.4. FUS-Cav Treatment Immediately Induced Sonoporation Effect

**Figure 6:**
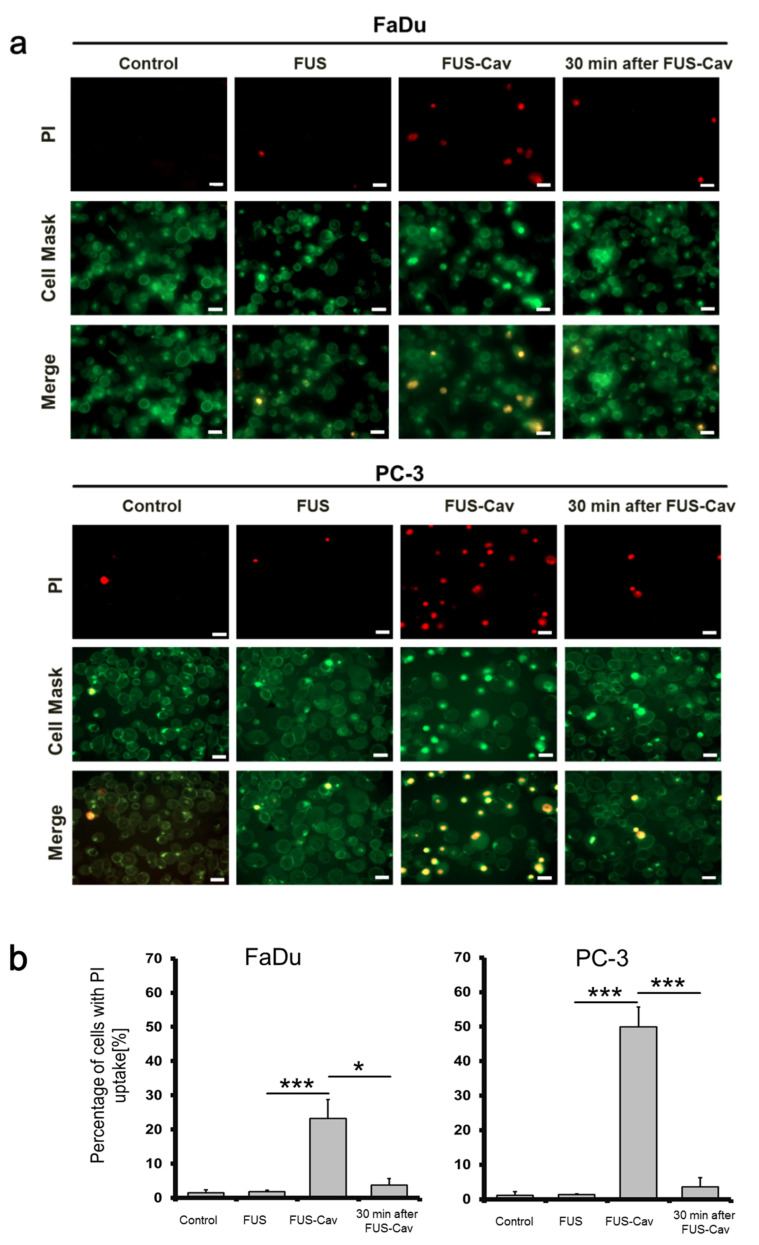
FUS-Cav induces sonoporation. FaDu and PC-3 cells were treated by FUS-Cav treatment at 1136 W/cm^2^ and with propidium iodide (PI) as a marker during and 30 min after treatment. (**a**) Representative fluorescence microscopy images showed an increase in red PI fluorescence during FUS-Cav. PI-stained cell nucleus (red) and CellMask-stained cell membrane (green), scale bar = 30 µm. (**b**) Semi-quantitative result of PI-positive percentage representing the occurrence of sonoporation. Data normalized to total cell number as 100%, n = 6, * *p* ≤ 0.05; ** *p* ≤ 0.01; *** *p* ≤ 0.001.

The sonoporation effect after FUS-Cav treatment was exemplarily investigated in FaDu and PC-3 cell lines, intracellular uptake of PI was considered as an indicator of sonoporation, CellMask^TM^ staining was employed for visualization of the cell membrane. Since the dimension of the focal field covered one well of the 96-well plate, the fluorescent images were taken randomly in the wells. FUS-Cav treatment at the intensity of 1136 W/cm^2^ with a total sonication duration of 40 s led to an increased percentage of intracellular PI in FaDu and PC-3 cells and suggested the occurrence of sonoporation effect. In contrast, FUS treatment at an intensity of 129 W/cm^2^ showed limited sonoporation events compared to the control. Fluorescence microscopy images demonstrated that cell membrane stained by CellMask^TM^ was not significantly changed before and after FUS-Cav treatment (Figure 6a). Remarkably, the percentage of PI-positive cells was significantly enhanced to 49.9% in PC-3 and 23.3% in FaDu cells (Figure 6b) immediately after exposure to FUS-Cav and only 4% PI-positive cells were observed in both cell lines 30 min post-treatment.

## 4. Discussion

Most of the pre-clinical studies focused on the application of FUS-induced thermal effects (ablation or hyperthermia) for the treatment of various cancer types in combination with chemo- or radiation therapy [7,31]. The mechanical effect of FUS attracted more interest in recent years for histotripsy, thrombolysis and sonodynamic therapy [32]. Ultrasound-induced cavitation is a promising and fast technology for induction of necrosis [33], reduction of localized fat [34], support drug/gene delivery [35], applied for thrombolysis [36] and opening of the BBB [37]. Even cavitation demonstrated good potential in cancer therapy, the effect of the combination of cavitation and other therapies was not sufficiently investigated and the underlying mechanisms remain unclear. Moreover, cavitation is avoided in most clinical HIFU treatments (e.g., thermal ablation) to prevent the collateral damage of healthy organs. Furthermore, in MR-guided HIFU systems such as Sonalleve (Profound medical) and Exablate (Insightec) systems, the thermometry is affected by cavitation and lead to an immediate stop of a running HIFU treatment. Because cavitation is not a robust reliable occurring phenomenon, lack of control techniques is the main challenge in current clinical HIFU applications [38]; however, various sonication schemes were reported for cavitation controlling and may play an essential role to harness clinical cavitation treatment [39]. The development of clinical HIFU lithotripsy systems provides a novel strategy for a controlled cavitation activity with dual-frequency waves. Cavitation clouds are created by high-frequency ultrasound (1–4 MHz) and are forced to collapse with a lower frequency at 0.5 MHz [40]. In addition, the presence of the ultrasound contrast agents, microbubbles, allows for the induction of a reproducible cavitation effect, especially in BBB opening and the alteration of vessel permeability to support drug delivery. The safety and clinical feasibility of cavitation have been investigated in recent clinical trials for the treatment of brain diseases [41,42], showing no detectable adverse effects. More clinical trials are needed to accumulate the understanding of FUS-induced cavitation for cancer therapies and in combination with other therapeutic modalities as well. Cavitation dose measurement was conducted in many studies by broadband noise measurement with a hydrophone, acoustic emission, bubble observation, sonochemical luminescence, and aluminum foil erosion [43]. It is challenging to estimate cavitation thresholds by numeric simulation because of the nonlinear influence of acoustic parameters. Hence, the measurement with a hydrophone is the most commonly used method so far. The definition of the cavitation threshold is controversially discussed in literatures based on different experimental setup [44].

The cavitation threshold was defined as three-fold the SD of unsonicated samples in our study as reported previously by Wu et al. [45]. In our experiment, the results were chemically validated by assessing the generation of hydroxyl radicals (·OH) with the TA method to confirm the inertial cavitation dose measured with the fiber-optic hydrophone. The cavitation threshold was defined here as the intensity at 344 W/cm^2^ and above; this is consistent with the report of Brüingk [46] that cavitation occurred at intensities above 200 W/cm^2^.

Cavitation dose was measured with the same FUS setup used for biological experiments using the in vitro setup in 96-well plates. This allows for analyzing the correlations between acoustic waves and biological interactions in cells. In order to monitor the real-time temperature without disturbing acoustic field, it has so far not been possible to avoid reflections of acoustic waves at the medium/air interface and likely standing waves as well in the described in vitro setup. However, the influence of reflection might not lead to dramatic differences in cavitation assessment. As reported by Robertson et al., the existence of acoustic reflections only led to a 15% lower inertial cavitation dose [47]. A possible impact of reflections and standing waves on cells is a limitation of this study, and the acoustic intensity for the induction of cavitation needs further optimization and validation using an in vivo model.

To separate the thermal effect and cavitation, the activation of ultrasound was controlled by the LabView program with a feedback loop to the thermal camera, and the ultrasound was deactivated when the temperature reached 39 °C. The intensity of FUS and FUS-Cav resulted in different temperature profiles, and temperature increased much faster at a higher intensity, consequently leading to different activation durations in each sonication cycle and total treatment duration. Additionally, due to the technical limitation, there is no commercially available system which can simulate the rapid dynamic temperature changes on monolayer cells during FUS exposure. The combination of HT and RT reveals a significant reduction in cell clonogenic survival compared to single RT indicating the radiosensitization effect of conventional HT as reported in the literature [48]. FUS and FUS-Cav alone showed a limited impact on the reduction of clonogenic survival and metabolic activity, demonstrating that single FUS and FUS-Cav are less harmful to cancer cells compared to HT. A combination of FUS-induced cavitation and RT showed a significant reduction of cell clonogenic survival compared to single treatment groups in all cell lines indicating the radioadditive effect of cavitation. Interestingly, short high-intensity FUS-induced cavitation treatment demonstrated a comparable radioadditive effect to HT at 45 °C for 30 min in FaDu cells, indicating that the equivalent radiosensitization effect of HT could be achieved by using short FUS with cavitation, and the treatment duration could be remarkably reduced from 30 to 2 min. Higher effects of FUS-Cav + 10 Gy on the reduction of both cellular metabolic activity and cell invasion compared to single treatment indicating FUS-Cav is an effective tool to increase the effect of RT. It has previously been shown that a combination of ultrasound and radiation can enhance the antitumor and antivascular effect with the presence of microbubbles in vivo [49]. The impact of ultrasound on cell division capability, ultrastructural changes, chromosomal and cytogenetic effects and functional changes supposed to be the potential mechanism for the sensitization of cancer cells to radiation was reported [50]. Localized cavitation for 2–3 min was reported to sensitize hepatocellular carcinoma to radiotherapy by improving tumor responses in an orthotopic rat model [51]. Moreover, the major clinical problem of head and neck cancer is the high resistance to radiation, and the systemic toxicity of chemotherapy may induce several adverse effects. Thus, there is an urgent need in this tumor entity to overcome the radioresistance by lowering the systemic toxicity [52]. Our finding offers an alternative strategy to sensitize cancer cells to RT non-invasively by the application of short FUS-induced cavitation shots, especially for head and neck cancer and prostate cancer.

HT in the range of 39–45 °C was reported to arrest cell proliferation, kill cancer cells, and induce heat shock proteins to suppress tumor growth. HT is typically applied in combination with other treatment modalities in the clinic [53]. Santos et al. reported that cavitation can enhance the effect of ultrasound-induced hyperthermia [54]; however, a combination of cavitation and other HT modalities was not reported in the literature so far. In this context, we combined short FUS treatments with HT to investigate the synergistic effect of these two treatment modalities. A combination of FUS or FUS-Cav treatment and HT result in a greater reduction of cell clonogenic survival in head and neck and prostate cancer cell lines compared to single HT indicating an additive effect of combined short FUS shots and HT. FUS-Cav + HT induced greater loss of clonogenic survival than FUS + HT in PC-3 cells. This suggests that the cavitation effect of FUS might be more responsible for the effects in PC-3 cells. It is notable here that FUS-Cav and HT results in a reduction of metabolic activity and loss of the cell invasion capability. This finding is consistent with the report that a combination of FUS and RT may lead to the deformation of the cell membrane [55]. We hypothesize that exposure to FUS-Cav initially changed cell membranes and induced the reduction of cell metabolic activity [56] followed by a loss of invasion ability as a consequence.

PI is a fluorescent dye with a molecular diameter of 0.8 nm that was reported by Wamel et al. to investigate the sonoporation effect in vitro [57]. The pores generated by sonoporation were reported with a size of 110 ± 40 nm [58], allowing for the penetration of PI molecular through cell membrane. Exposure to FUS-Cav with high intensities resulted in a 10–20% reduction in cell confluency compared to untreated cells. The results were quantified as a percentage of PI-positive cells to avoid the influence of detachment. The percentage of PI-stained cells was increased immediately post-FUS-Cav treatment in both head and neck and prostate cancer cell lines representing the enhanced uptake of PI, whereas the low percentage of PI-positive cells 30 min after treatment suggests low cell death and the integrity of cell membrane. Even sonoporation is a general phenomenon in cells after FUS exposure, and the observed extent of sonoporation is different between cell lines; specifically, more PC-3 cells were affected by sonoporation compared to FaDu cells. Sonoporation was reported to induce apoptosis [59], cell cycle arrest and the inhibition of DNA damage repair [60], which could be the potential mechanism for the reduction of cell clonogenic survival. We hypothesize that the cell membrane was disrupted due to the occurrence of sonoporation immediately after sonication, but the disrupted cell membrane closed 30 min post-sonication and PI is not able to penetrate the cell membrane, indirectly hinting that the sonoporation effect is reversible. It is consistent to the report from Yang that the cavitation-induced sonoporation is reparable via cell self-sealing in MCF-7 cells [61], which is supposed to be the biophysical mechanism here leading to lower survival of PC-3 compared to FaDu.

The present study shows the potential of FUS-induced cavitation as a sensitizer to RT and HT using an in vitro cell culture model. The results need further validation with an in vivo study where optimization of the ultrasound parameters according to the ultrasound propagation properties of tissues is necessary. Nonetheless, our in vitro study shows the first evidence that FUS-Cav sensitize cancer cells to RT and HT, and gives a good start point for further in vivo and clinical studies. Administration of microbubbles should be considered to generate constrained cavitation effects with reduced acoustic intensities in animals or humans. Detailed biological investigations at the cellular and molecular level regarding apoptosis, DNA damage and cell cycle will be performed to complete the understanding of underlying mechanisms.

## 5. Conclusions

Our findings demonstrate short-term and long-term additive effects of FUS (short FUS with or without cavitation) to RT or HT at the cellular level which was displayed by a reduction in metabolic activity and clonogenic survival. The combination of short FUS with cavitation (40 s) and RT leads to a comparable radiosensitization effect as HT at 45 °C for 30 min. Therefore, the treatment duration could be remarkably reduced from 30 to 2 min, especially for head and neck cancer cells. A decrease in cell invasion capability and induction of sonoporation are supposed to be the mechanisms of sensitization. Our results suggest that short FUS treatment could be an efficient tool to enhance the effect of RT or HT precisely and non-invasively and may provide a chance for less invasive adjuvant therapy in the future.

## Supplementary Materials

The following are available online at https://www.mdpi.com/2073-4409/9/12/2595/s1, Figure S1: Impact of various hyperthermia duration and radiation dose on cell metabolic activity. Cancer.

## Abbreviations

FUS	Focused ultrasound
RT	Radiation therapy
HT	Hyperthermia
FUS-Cav	FUS shot with cavitation
HIFU	High-intensity focused ultrasound
MR	Magnetic resonance
BBB	Blood–brain barrier
PCD	Passive cavitation detector
TA	Terephthalate acid
FOH	Fiber-optic hydrophone
DMEM	Dulbecco’s Modified Eagle’s Medium
HEPES	4-(2- Hydroxyethyl)-piperazine- 1- ethanesulfonic acid
MEM	Minimum Essential Medium
FBS	Fetal bovine serum
PBS	Phosphate-buffered saline
EDTA	Ethylenediaminetetraacetic acid
FFT	Fast Fourier transformation
RMS	Root-mean-square
SF	Survival fraction
PI	Propidium iodide
SD	Standard deviation
SEM	Standard error of the mean
