# Supplementary material for: Focused Ultrasound-Induced Cavitation Sensitizes Cancer Cells to Radiation Therapy and Hyperthermia
Source: Cells. 2020 Dec 3;9(12):2595. doi: 10.3390/cells9122595 (PMC7761886; doi:10.3390/cells9122595)
Supplement: Supplementary file 1 [file cells-09-02595-s001.pdf]

Supplementary

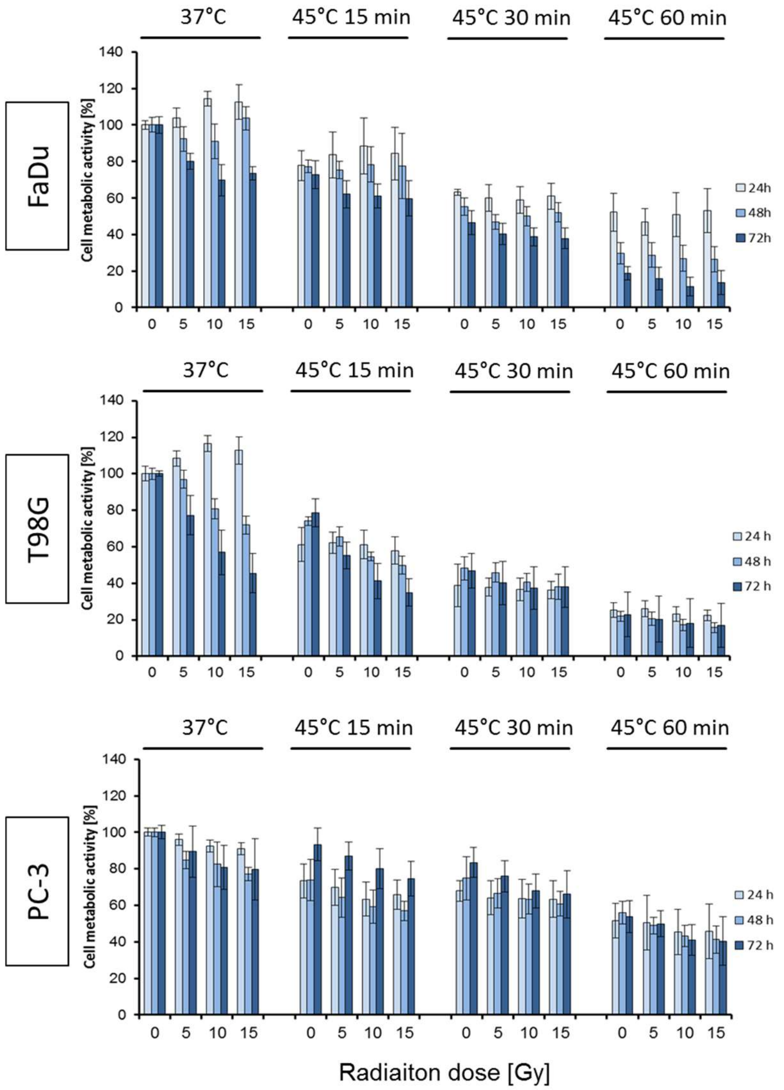

Figure S1. Impact of various hyperthermia duration and radiation dose on cell metabolic activity. Cancer cells were treated with water bath-HT at 45 °C for 15, 30 and 60 min followed by single dose irradiation at 5, 10 and 15 Gy. Relative cellular metabolic activity was measured with WST-1 assay. Data were normalized to untreated control, which were set as 100 %. n = 9
